# Personality, predation and group size: unravelling behavioural drivers of lionfish (*Pterois volitans*) invasion success

**DOI:** 10.1098/rsos.251158

**Published:** 2025-11-05

**Authors:** Monica McCard, Karla Alujević, Nathan McCard, Gareth Arnott, Louise Kregting, Jaimie T. A. Dick, Josie South

**Affiliations:** ^1^ Institute for Global Food Security, School of Biological Sciences, Queen's University Belfast, Belfast, Northern Ireland, UK; ^2^ Queen's University Belfast Marine Laboratory, Portaferry, Northern Ireland, UK; ^3^ School of Biological and Environmental Sciences, Liverpool John Moores University, Liverpool, UK; ^4^ Department of Biology and Program in Ecology, Evolution, and Conservation Biology, University of Nevada Reno, Reno, NV, USA; ^5^ The New Zealand Institute for Plant and Food Research Ltd, Nelson, New Zealand; ^6^ Water@Leeds, School of Biological Sciences, University of Leeds, Leeds, UK

**Keywords:** personality, functional response, invasive species, predator-prey interactions

## Abstract

Global biodiversity is in rapid decline, with invasive alien species playing a major role. Predicting which is most damaging and under what conditions is key to proactive management. We investigated whether behavioural traits, specifically boldness and exploration, predict ecological impact in the invasive red lionfish (*Pterois volitans*). Despite the modest sample size of adults (*n* = 8) and juvenile (*n* = 8) lionfish, using repeated behavioural assays, we found strong personality consistency: 93% of juveniles and 56% of adults used shelter, with traits like latency to interact with novel objects showing high repeatability. Bold individuals spent less time in shelter and interacted more with novel stimuli. However, in groups of eight, personality expression shifted, with only 7% of juveniles and 44% of adults using shelter, indicating that social context alters behaviour. Functional response experiments revealed Type II feeding curves across three prey species, reflecting a saturating, hyperbolic relationship in which predators rapidly consume prey at low densities but are increasingly constrained by handling time as prey density rises. Neither adult nor juvenile lionfish reduced feeding effort when prey became scarce, allowing them to exert strong predation pressure even at low prey densities. Adults displayed significantly higher attack rates and shorter handling times on *Artemia salina*, whereas juveniles showed these patterns towards *Gammarus oceanicus*, underscoring the greater *per capita* feeding impact of adults. Contrary to expectations, boldness did not correlate with feeding impact but was linked to slower reaction times in shy individuals. These findings highlight the complex, context-dependent relationship between personality and ecological impact during invasions.

## 1. Introduction

Translocation of species beyond their native range due to increasingly connected transport networks is a defining feature of the Anthropocene [1]. Invasive alien species are recognized as key drivers of biodiversity loss [2]. Determining which traits are attributed with successful invasive non-native species is a priority to predict future invasions [3]. Traits such as high fecundity, generalist feeding habits and broad physiological tolerance to environmental conditions are widely recognized as contributing to invasion success [3–6]. However, these traits are assessed at the species level and are most useful for predicting invasions in areas where the species has not yet become established. Once established, management must operate at the population level, where invasion success depends on local environmental context and time since introduction [7–9].

Selection processes acting on the invasive population include spatial sorting, density dependence and environmental filtering; which act on morphological traits [10,11]. However, invasion barriers can also act as a filter on behavioural traits [12]. Wherein invasive populations may demonstrate differences in boldness and exploratory behaviour compared to native or domestic populations [4,12]. These trait filters may be lost or gained depending on time since invasion, for example individuals in recently introduced populations of round goby (*Neogobius melanostomus*) were more active and faster to disperse compared to individuals from older invasions [13]. Although behavioural traits were not directly assessed in this study, personality was examined. Personality refers to consistent individual differences in behaviour across time and contexts [14]. When such behaviours are both repeatable and correlated, they constitute what is termed a behavioural type [15]. For example, individuals often exhibit consistent differences in personality such as boldness, exploratory behaviour, or aggression, with some displaying a ‘bold’ personality type while others are more ‘shy’ These personality variations can shape how individuals engage with their environment and may influence ecological processes like predation [16]. In the context of invasive species, assessing whether subsets of invasive groups exhibit diverging personalities, and whether some are correlated with indicators of high consumptive ecological impact (e.g. feeding rate, attack rate or clearance rate), will help to prioritize stages of the invasion gradient for targeted management. Such behavioural filtering could reveal high-impact individuals within established populations, offering a finer-scale approach to mitigating overall invasion progress and ecological damage.

Linking personality to ecological impact has proven challenging, as relationships are often species, or population, specific. For example, invasive Siamese fighting fish (*Betta splendens*) showed higher activity but lower feeding rates than domestic individuals [17], whereas aggressive signal crayfish (*Pacifastacus leniusculus*) had higher feeding rates [11]. In some species, invasion front populations exhibit reduced boldness but greater feeding impact [18], while in others, less bold individuals show enhanced growth and fitness [19]. Moreover, personality effects are context dependent: social dynamics such as competition and facilitation can change individual behaviour and alter ecological outcomes [20,21]. This highlights the importance of testing personality expression across different social contexts, since group dynamics such as competition or facilitation can shift individual behaviour and thereby alter ecological outcomes. Linking these behavioural traits to feeding performance is essential for understanding invasion impacts.

Functional response (FR), the relationship between prey density and the rate at which a predator consumes prey, is a widely used metric to assess the ecological impact of an invasive species [22]. It integrates both behavioural and physiological traits and provides a scalable framework to estimate *per capita* effects, especially when comparing individuals or groups with differing behaviour/personalities [23,24]. Higher attack rates and lower handling times are indicative of greater ecological impact, and FR analysis has been used to prioritize management of high-impact invaders [23,25]. In this context, linking personality variation (e.g. boldness, exploration) with variation in FR parameters offers a powerful approach to understand and predict context-specific invasion impacts.

The red lionfish, *Pterois volitans*, hereafter referred to as ‘lionfish’, provides an ideal model to test these links. Native to the Indo-Pacific region, lionfish have become highly successful invaders throughout the western Atlantic, Caribbean, Gulf of Mexico [26,27] and the Mediterranean seas [28,29]. Their invasion success has been attributed to traits common among invasive alien species, including high fecundity [30], large body size [31], dispersal ability [32,33] and generalist diet [16,34]. However, culling, the primary management tool, may inadvertently select against bold or exploratory individuals, shifting personality composition within populations [35,36]. Such selective pressures could alter both individual ecological impact and group-level dynamics, yet this remains unexplored. Here, we test whether lionfish exhibit consistent personality traits and whether these traits predict ecological impact. Specifically, we hypothesize that: (i) lionfish display repeatable individual differences in behaviour indicative of personality; (ii) individuals with bolder or more exploratory personalities will have higher ecological impact, as measured by FR metrics and (iii) the expression and ecological relevance of these behavioural traits are modulated by life stage and group size (i.e. social context), potentially affecting the predictability of ecological impact.

## 2. Material and methods

### 2.1. Animal collection and maintenance

All lionfish were purchased from Seahorse Aquarium, Dublin. These individuals originated from wild-caught lionfish from the western Atlantic invasion range, which were subsequently bred in captivity by a private breeder before being supplied to the aquarium. Species confirmation was obtained retrospectively through dissections and genetic tests post-experimentation, which confirmed all individuals as *P. volitans*. Experiments were undertaken at Queen’s University Marine Laboratory, Portaferry, Northern Ireland, between October 2017 and September 2018. Juvenile lionfish (*n* = 8) had a total body length (mean ± SE) of 100.2 ± 3.7 mm, with a pectoral fin diameter of 57.9 ± 4.8 mm, as measured across the widest point when elongated. Adults (*n* = 8) measured 322 ± 7.9 mm in length with a pectoral fin diameter of 265.5 ± 6.4 mm. Each lionfish was classed as adult/juvenile and assigned a unique number ranging from 1−8 based on their markings, colouration and/or specific differences (see S.1). Juveniles were kept together in a holding tank (W: 32 cm × L: 152.4 cm × H: 45.7 cm, 220 L), while adults were housed two adults per tank (W: 82.3 cm × L: 228.6 cm × H: 61 cm, 1146 L). Holding tanks had the same filtration set up, external filtration containing UV- and sand-filtered recirculating Strangford Lough seawater. Water was changed daily by 25% and tested daily for water chemistry properties (pH, NH_4_), and temperature maintained using an aquarium heater under a 16 :8 hour light-dark regime. The temperature was maintained at 25 ± 1.0°C. Lionfish were fed daily *ad libitum* on frozen anchovy to avoid predator learning behaviour to the focal experimental prey species.

### 2.2. Behavioural assays—novel object

Novel object assays were used as measures of boldness and exploratory behaviour. Each fish was exposed alone (*n* = 3) and in a group of eight individuals (*n* = 3 for each individual), resulting in six exposures per fish. Assays were performed over a 21-day period with a 2-day break between exposures and with the tanks cleaned between each use. Tank size changed with size of lionfish (juveniles: W: 33 cm × L: 45.7 cm × H: 30.5 cm, 45 L; adults: W: 50.8 cm × L: 132.1 cm × H: 38.1 cm, 255 L) for individual experiments and then for group experiments (juveniles: W: 32 cm × L: 152.4 cm × H: 45.7 cm, 220 L; adults: W: 254 cm × L: 457.2 cm × H: 88.9 cm, 10 000 L). A small shelter was added into the tanks prior to experiments (plastic pipe – W: 10.5 cm × L: 21 cm × H: 6 cm). Experimental tanks were scaled to reflect the difference between juvenile and adult lionfish when pectoral fins were fully elongated during feeding trails, where adult lionfish were around five times the size of juveniles. To record behavioural responses, two GoPro^®^ Hero10^®^ cameras were mounted on the top of tanks using a wide field of view, with black out sheets to cover the sides of the tanks to reduce external stimuli. Fish were acclimated to the experimental arena for 30 minutes and then presented with a randomly selected small toy figure as it was lowered into the tank (see electronic supplemenary matrial, S.2) for a 10-minute period. Recordings were then reviewed by the same observer using BORIS Software [37], and personalities were categorized into two main traits: exploration/shyness (measured by latency to contact the novel object, time spent in shelter and frequency of shelter visits) and boldness (measured by the number of contacts with the novel object). These personalities were assessed using the ethogram in table 1, enabling the classification of individuals along a bold-shy continuum (table 2).

**Table 1.** Ethogram showing behaviours measured (in seconds) for all lionfish during the novel object experiments.

behaviour	number	description
contacting the object	1	latency to contact the object (time taken to touch the object)
hits of the object	2	how many times the object was touched (either part of the lionfish body or fins)
shelter time	3	time spent in the shelter (duration of time where the body of lionfish is fully in the shelter)
times at shelter	4	number of times the lionfish went to the shelter

**Table 2.** Definition with associated description of boldness and shyness used in this study.

category	description
boldness	where the lionfish spends a longer period of time at the novel object with minimal time spent in the shelter.
shyness	this is indicated by the lionfish having spent no time at the novel object and most of the time spent in the shelter.

### 2.3. Functional response procedure

Feeding experiments were conducted within glass tanks (juveniles: W: 33 cm × L: 45.7 cm × H: 30.5 cm, 45 L; adults: W: 50.8 cm × L: 132.1 cm × H: 38.1 cm, 255 L) also maintained at 25.0 ± 1.0°C, and all fish were acclimated in the experimental arenas 30 minutes prior to experimentation. Prey species used for the FR experiments were all live and consisted of marine gammarid (*Gammarus oceanicus),* dwarf white shrimp (*Palaemonetes varians*), and brine shrimp (*Artemia salina*). Prey were purchased from Grosvenor Tropicals, Lisburn and maintained under identical conditions to the predators in separate holding tanks (W: 15.2 cm × L: 20.32 cm × H: 17.8 cm, 10 L). All prey species were easily available and found in high quantities (see [38] for dietary importance of crustaceans for lionfish). In this case, *G. oceanicus* represents a benthic crustacean, i.e. amphipods and isopods found in lionfish diets in invaded ranges [39]. *Palaemonetes varians* represents a palaemonid shrimp species abundant across lionfish invaded ranges found in lionfish diets [40], while *A. salina* represents a small pelagic crustacean prey [41]. The prey used here do not currently overlap with lionfish distributions but are used as functional proxies [42,43]. Intraspecific prey size was standardized throughout all trials, including all prey used (total length mm ± SE: *G. oceanicus* 10.3 ± 1.2 mm; *P. varians* 10.7 ± 0.3 mm; *A. salina* 6.6 ± 1.1 mm). All necessary ethical protocols were complied with throughout the experimental process after being sought from the School of Biological Sciences ethics committee, Queen’s University Belfast.

Adult lionfish were provided with each prey species individually at 10 different densities (2, 4, 8, 16, 32, 64, 128, 256, 512, 1024; *n* = 6 per prey species, per density), whereas juvenile lionfish prey were supplied at 13 densities (2, 4, 8, 16, 20, 25, 30, 35, 40, 45, 50, 55, 60; *n* = 6 per prey species, per density) following a randomized pattern. Prey were introduced, and the lionfish were allowed to feed for 3 hours and then prey left alive were counted. Initial reaction times of lionfish to first successful attack was recorded in each instance using a stopwatch. Control groups were included, consisting of one replicate of each prey type across all densities in the absence of lionfish.

### 2.4. Statistical analyses

All statistical analyses were conducted in the R programming environment [44].

### 2.5. Repeatability

To test if lionfish showed consistent inter-individual differences across contexts, we estimated repeatability as ratios of between-individual phenotypic variance to total phenotypic variance in our sample [45]. For personalities obtained during the novel object trials (latency to contact the object, time spent in shelter, number of times at shelter, and number of contacts with the object), we tested whether individual lionfish showed consistent responses when presented with each of the different novel objects, both when tested individually and in a group setting. Repeatability was calculated using mixed-effects models fitted with novel object and age group as fixed predictors and lionfish identity as random effect [46,47]. Similarly, we tested whether individual lionfish FR (maximum feeding rate and reaction time) was consistent across prey type (*G. oceanicus, P. varians* and *A. salina*), where prey type and age group were set as fixed predictors and lionfish identity as random effect. Linear mixed-effects models were run using the ‘lmer’ and ‘glmer’ functions (‘lme4’ package). Confidence intervals (95% CI) were calculated using a parametric bootstrapping method within the LMM approach [48]. Results closer to 1 indicate high repeatability while those closer to 0 indicate lack of repeatability. All statistical analyses were performed in R Studio [44] for R 4.2.0.

### 2.6. Principal component analysis

Principal component analysis (PCA) was used to reduce the dimensionality of behavioural data obtained during the novel object trials and test for the presence of personality traits. Principal components were retained following the Kaiser-Guttman criterion (eigenvalues >1; [49]). The eigenvectors consider personality parameters showing the variation in the data that could be explained (PC1) along with another level of variation that is significant (PC2). Positive or negative values over 0.4 show that trait has contributed significantly to the variation in that component [50,51]. We therefore used individual PC scores obtained from PCA analyses as a measure of individuals’ personality types to estimate how lionfish personality changes when lionfish are alone (single fish trials) versus in groups.

### 2.7. Correlations between personality and functional responses

To test whether personality is predicted by maximum feeding rate and feeding reaction time, linear models with personality PC score of individual and group testing (PC1 and PC2 separately) were fitted as a dependent variable and size, maximum feeding rate and reaction time as fixed effects. A Type II *post hoc* was used to determine differences between groups as there was no significant interaction term.

### 2.8. Functional response

FRs were categorized into Type II or Type III using a binomial logistic regression via *frair::frair_test*, where Type II responses are indicative of high consumption at low prey densities and commonly predict high-impact invasive species, and Type III responses are characterized by a sigmoidal relationship between consumption and density where there is a low-density prey refugia. Type II responses were modelled with Rogers random predator equation ([52]; equation (2.1)) and Type III responses were modelled with Hassell’s Type III equation (2.2), both of which account for non-replacement of prey.


(2.1) $$N_{e}=\;N_{0}\left(1-ex\left(a\left(N_{e}h-T\right)\right)\right),$$

wherein *N*_e_ is the number of prey eaten, *N*_0_ is the initial prey density, *T* is the total time available and *a* and *h* are the mechanistically interpretable coefficients for attack rate and handling time, respectively. Whereas for Type III:


(2.2) $$N_{e}=N_{0}\left\{1-ex[(d+bN_{0}(hN_{e}-T)/(1+cN_{0})]\right\}.$$

Here, *a* is a hyperbolic function of *N*_0_ [53], *b* denotes the attack rate, *c* is a constant that influences the sigmoidal shape of the response (associated with prey refuge or switching behaviour) and *d* represents a baseline predation rate or mortality factor. FR data for each individual and prey type were then non-parametrically bootstrapped (*n* = 2000) to generate 95% confidence intervals. These intervals were based on the initial maximum likelihood estimates of parameters '*a*' and '*h*', which were obtained using *frair::frair_fit*. Maximum feeding estimates were then calculated as 1 */h*.

## 3. Results

### 3.1. Prey survival

Across control groups for all prey species, survival exceeded 99% in the absence of lionfish. Therefore, all mortality was assumed to be due to predation in the FR experiments.

### 3.2. Behaviour assays

Overall, 93% of juvenile and 56% of adult lionfish utilized the shelter when trials were conducted on individuals separately. However, when in groups, this occurred markedly less for juvenile lionfish where 7% utilized the shelter and only a small decrease to 44% of adult lionfish utilized the shelter in groups.

### 3.3. Repeatability

Both adult and juvenile lionfish showed consistent inter-individual differences in behaviour when exposed to the different novel objects, both in individual and group trials. Latency to contact a novel object, time spent in the shelter and number of hits of object were highly repeatable with estimates ranging from 0.86 to 0.96 in single fish trials and 0.52 to 0.99 in group trials (table 3). Individuals showed comparatively lower consistency in the number of times they visited the shelter (R = 0.43 in single fish trials and R = 0.34 in group trials). In FR trials, lionfish showed highly consistent individual responses for feeding reaction time (at highest prey density; R = 0.95) but lower consistency in maximum feeding rate (R = 0.28) across prey types.

**Table 3.** Repeatability estimates (*R*) and standard errors (SE) for inter-individual variation in lionfish feeding responses (FR) and personality trait responses when faced with novel objects (NO). Repeatability estimates are reported in the range from 0 to 1, where 0 represents no repeatability and 1 represents complete repeatability.

	test	trait	*R*	95% CI lower	95% CI upper
single fish	NO	time to reach object	0.957	0.896	0.983
	NO	time spent in shelter	0.960	0.903	0.985
	NO	number of times at shelter	0.434	0.159	0.596
	NO	number of hits of object	0.859	0.581	0.963
groups	NO	time to reach object	0.808	0.602	0.918
	NO	time spent in shelter	0.991	0.978	0.996
	NO	number of times at shelter	0.338	0.005	0.527
	NO	number of hits of object	0.517	0.222	0.660
	FR	maximum feeding rate	0.283	0.000	0.606
	FR	reaction time (at highest prey density)	0.947	0.877	0.978

### 3.4. Principal component analysis

**Figure 1.**
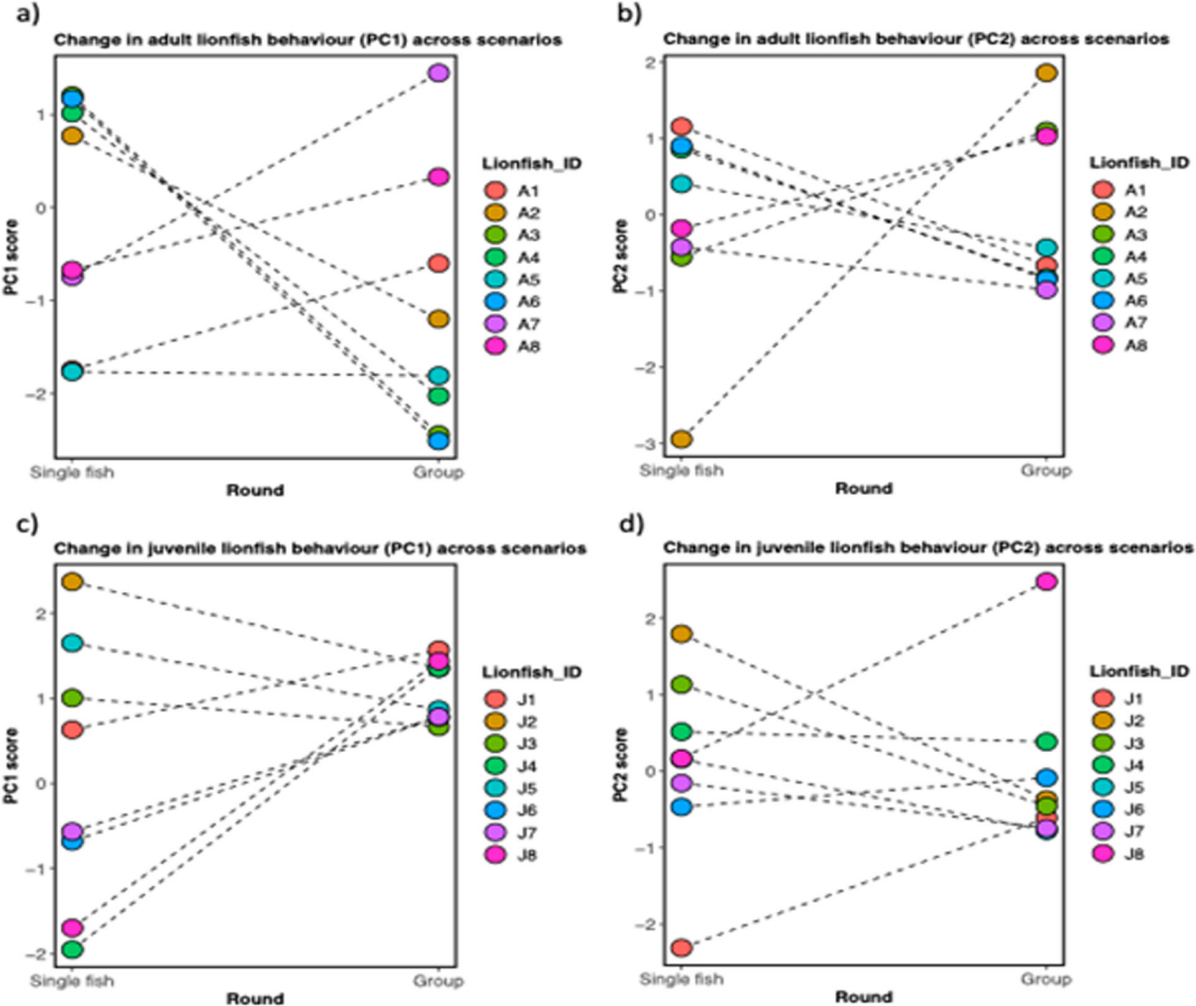
Change in personality traits across scenarios (single animal versus group) during novel object experiment for (a) adult lionfish (*Pterois volitans*) PC1 and (b) adult lionfish PC2 and (c) juvenile lionfish PC1 and (d) juvenile lionfish PC2. The eigenvectors represented personality parameters, with the first principal component (PC1) capturing the primary source of variation in the data and the second component (PC2) accounting for an additional, statistically significant dimension of variation.

Principal component analyses for personality traits measured during novel object single fish trials showed that the first axis (PC1) explained most of the total variance (45.2%), while PC2 explained 35.3% (table 4). Here, PC1 showed significant shifts in individual personalities when comparing their responses in isolation versus group settings. In isolation, bolder lionfish exhibited personality traits characterized by greater exploration and less time spent in shelter, while shyer individuals were more reserved, spending more time in the shelter and showing less interest in novel objects. However, when housed in groups of eight, the personalities of both the bolder and shyer lionfish appeared to change. In the group setting, bolder individuals exhibited more inhibited personalities, displaying less exploration of the novel object compared to their behaviour in isolation. In contrast, the shyer individuals displayed a shift towards bolder personalities, showing increased exploration and a reduced tendency to seek shelter. Specifically, shyer lionfish spent less time in the shelter when in groups than they did when isolated (see figure 1). The presence of conspecifics appears to influence personality expression, with bold individuals becoming more reserved and shy individuals showing increased confidence and exploratory behaviour in a group context. For personality traits measured during novel object group trials, the first axis (PC1) explained 53.7% and PC2 26.3% of the variance (table 4). Again, individuals that spent more time in the shelter hit the novel object less times; however, they visited the shelter more times (figure 1b,d).

**Table 4.** Component loadings of personality traits observed on two orthogonally rotated principal components (PC1 and PC2). Values highlighted in bold indicate behaviours that were considered to contribute to a component (loading of at least 0.4).

	behaviour	PC1	PC2
single fish	time to reach object	**0.810**	**0.657**
	time spent in shelter	**−0.779**	**0.608**
	number of times at shelter	**0.533**	0.285
	number of hits of object	**0.509**	0.259
	**% variance explained**	45.20	35.29
	**total variance explained**	80.49	
group	time to reach object	−0.381	**0.876**
	time spent in shelter	**0.894**	−0.144
	number of times at shelter	**0.690**	**0.495**
	number of hits of object	**−0.854**	−0.141
	**% variance explained**	53.72	26.33
	**total variance explained**	80.05	

### 3.5. Correlations between personality and feeding

**Figure 2.**
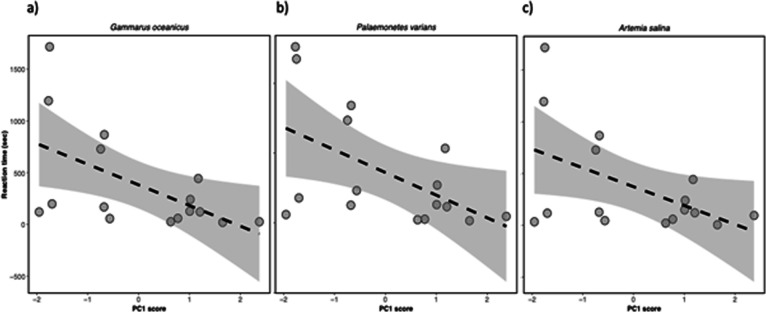
Correlations between personality traits and feeding reaction time of both juvenile and adult lionfish (*Pterois volitans*) towards prey species: (a) *Gammarus oceanicus*, (b) *Palaemonetes varians* and (c) *Artemia salina*.

Personality trait (PC1) was predicted by feeding reaction time (figure 2) but not maximum feeding rate for each of the three prey types (table 5). Size (in terms of length of lionfish) was included in initial models but did not have a significant effect on personality and was thus removed from the models during the model selection process. These results show that individuals that are shyer (i.e. spend more time in the shelter) have slower feeding reaction time but there is no correlation with maximum feeding rate.

**Table 5.** Parameters from the linear model testing for the effect of maximum feeding rate (MFR) and feeding reaction time (RT) on lionfish behavioural type (PC1). Significant values (*p* < 0.05) are in bold.

	predictor	β	SE	DF	t-value	*p*‐value
*G. oceanicus*	max feeding rate	0.003	0.002	13	1.44	0.173
	reaction time	−0.002	0.001	13	−2.82	0.015
*P. varians*	max feeding rate	0.003	0.002	13	1.51	0.155
	reaction time	−0.002	0.001	13	−2.74	0.017
*A. salina*	max feeding rate	0.001	0.001	13	1.66	0.122
	reaction time	−0.001	0.001	13	−2.43	0.030

### 3.6. Functional response

**Figure 3.**
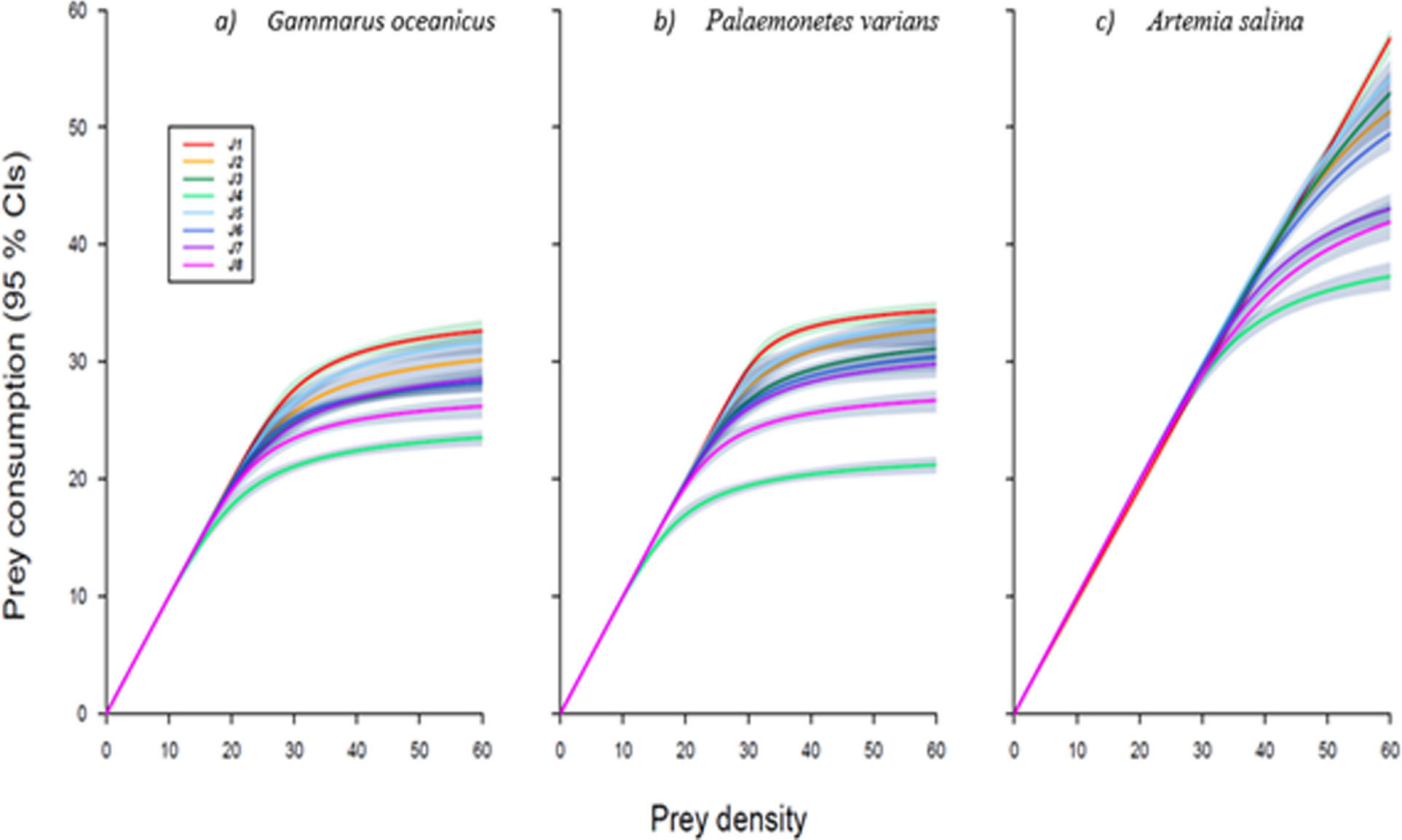
Functional responses showing consumption of prey with juvenile *Pterois volitans* (lionfish) when feeding as individuals (Lionfish 1−8). Towards prey species: (*a*) *Gammarus oceanicus*, (*b*) *Palaemonetes varians* and c) *Artemia salina*. Individual lionfish 2−8 produced a Type II functional response (FR) towards all prey, whereas lionfish 1 produced a Type III FR towards *A. salina*. Shaded areas are bootstrapped (*n* = 2000) 95% confidence intervals.

**Figure 4.**
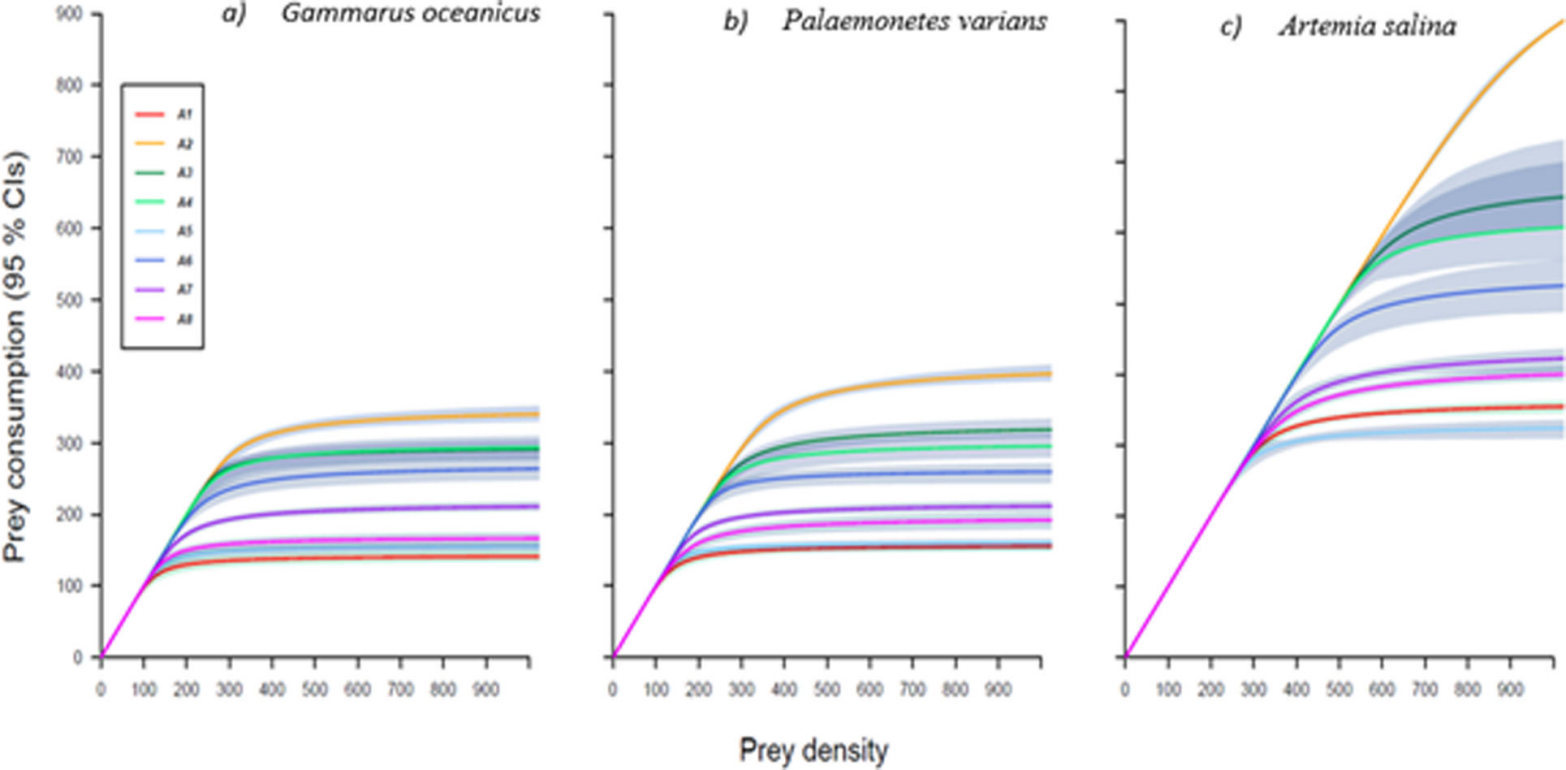
Functional responses showing consumption of prey with adult *Pterois volitans* (lionfish) when feeding as individuals (Lionfish 1−8). Towards prey species: (*a*) *Gammarus oceanicus*, (*b*) *Palaemonetes varians* and (*c*) *Artemia salina*. Individual lionfish all produced a Type II functional response (FR) towards all prey. Shaded areas are bootstrapped (*n* = 2000) 95% confidence intervals.

First-order terms for determining FR were significantly negative indicating Type II FR for all juvenile and adult lionfish towards *G. oceanicus* and *P. varians*. Whereas for *A. salina* a single juvenile lionfish had a first-order term which was significantly positive while the second-order term was significantly negative, indicating Type III. All other juvenile and adult lionfish had a significantly negative first-order term towards *A. salina*, indicating Type II FR (table 6 and 7, figures 3 and 4).

**Table 6.** First-order terms, functional response (FR) types, FR parameter estimates (*a*, *H,* and 1 */h*) with associated *p* values and standard error (se) results for both attack rate (*a*) and handling time (*h*) estimates for all prey species treatments for individual juvenile lionfish (J1-J8) *Pterois volitans* in which there were eight individual lionfish in total.

lionfish	prey	type	first-order terms	second-order term	attack rate**,** *a**,*** 95% CIs	handling time**,** *h***,** 95% CIs	*b*, 95% CIs	*c*, 95% CIs	maximum feeding rate (1 */h*)
J1	*G. oceanicus*	II	−0.086, <0.001		12.231, 8.475–23.250	0.028, 0.027–0.030			35.71
J2	*G. oceanicus*	II	−0.085, <0.001		9.282, 6.080–18.655	0.030, 0.028–0.033			33.33
J3	*G. oceanicus*	II	−0.074, <0.001		9.733, 7.062–14.213	0.033, 0.031–0.034			30.30
J4	*G. oceanicus*	II	−0.067, <0.001		7.350, 5.341–10.137	0.039, 0.038–0.041			25.64
J5	*G. oceanicus*	II	−0.079, <0.001		9.601, 7.022–14.178	0.029, 0.027–0.031			34.48
J6	*G. oceanicus*	II	−0.080, <0.001		11.596, 8.080–18.691	0.033, 0.032–0.035			30.30
J7	*G. oceanicus*	II	−0.078, <0.001		8.572, 6.371–11.857	0.032, 0.031–0.034			31.25
J8	*G. oceanicus*	II	−0.076, <0.001		9.760, 6.749–17.243	0.035, 0.034–0.038			28.57
J1	*P. varians*	II	−0.104, <0.001		23.051, 15.547–44.163	0.028, 0.027–0.029			35.71
J2	*P. varians*	II	−0.091, <0.001		13.181, 8.743–26.098	0.028, 0.027–0.030			35.71
J3	*P. varians*	II	−0.083, <0.001		11.185, 7.757–17.376	0.030, 0.028–0.031			33.33
J4	*P. varians*	II	−0.066, <0.001		7.742, 5.533–11.439	0.044, 0.042–0.047			22.73
J5	*P. varians*	II	−0.091, <0.001		13.462, 8.933–41.382	0.028, 0.026–0.030			35.71
J6	*P. varians*	II	−0.086, <0.001		11.274, 7.794–20.958	0.030, 0.029–0.033			33.33
J7	*P. varians*	II	−0.080, <0.001		11.131, 7.333–18.233	0.031, 0.028–0.033			32.26
J8	*P. varians*	II	−0.079, <0.001		11.222, 7.774–20.152	0.035, 0.033–0.037			28.57
J1	*A. salina*	III	0.192, <0.001	−0.002<0.001	—	0.028, 0.026–0.030	0.409, 0.400–0.421	0.029, 0.020 – 0.031	83.33
J2	*A. salina*	II	−0.120, <0.001		8.754, 6.359–12.358	0.015, 0.013–0.017			66.67
J3	*A. salina*	II	−0.094, <0.001		7.065, 5.353–9.623	0.013, 0.010–0.015			76.92
J4	*A. salina*	II	−0.102, <0.001		10.321, 7.562–16.166	0.024, 0.022–0.026			41.67
J5	*A. salina*	II	−0.120, <0.001		8.880, 6.120–13.604	0.013, 0.001–0.016			76.92
J6	*A. salina*	II	−0.108, <0.001		7.782, 5.752–10.836	0.015, 0.013–0.017			66.67
J7	*A. salina*	II	−0.108, <0.001		9.956, 7.313–14.142	0.020, 0.019–0.022			50
J8	*A. salina*	II	−0.106, <0.001		7.792, 6.008–10.386	0.020, 0.018–0.022			50

**Table 7.** First-order terms, functional response (FR) types, FR parameter estimates (*a*, *H* and 1 */h*) with associated *p* values and standard error (se) results for both attack rate (*a*) and handling time (*h*) estimates for all prey species treatments for individual adult (A1-A8) lionfish *Pterois volitans*.

lionfish	prey	type	first-order terms	attack rate, ***a,*** 95% CIs	handling time, ***h**,* 95% CIs	maximum feeding rate (1 */h***)**
A1	*G. oceanicus*	II	−0.003, <0.001	11.11, 6.720–22.898	0.006, 0.006–0.007	166.67
A2	*G. oceanicus*	II	−0.003, <0.001	14.25, 9.494–19.906	0.002, 0.002–0.003	500.00
A3	*G. oceanicus*	II	−0.003, <0.001	21.92, 10.570–38.507	0.003, 0.003–0.004	333.33
A4	*G. oceanicus*	II	−0.003, <0.001	16.99, 9.473–29.292	0.003, 0.003–0.003	333.33
A5	*G. oceanicus*	II	−0.003, <0.001	12.83, 7.070–27.925	0.006, 0.006–0.007	166.67
A6	*G. oceanicus*	II	−0.003, <0.001	11.29, 8.640–18.826	0.003, 0.003–0.004	333.33
A7	*G. oceanicus*	II	−0.003, <0.001	9.69, 7.177–13.244	0.004, 0.004–0.005	250.00
A8	*G. oceanicus*	II	−0.003, <0.001	12.28, 6.922–25.114	0.005, 0.005–0.006	200.00
A1	*P. varians*	II	−0.003, <0.001	10.37, 6.325–18.743	0.006, 0.006–0.007	166.67
A2	*P. varians*	II	−0.004, <0.001	12.58, 8.831–18.326	0.002, 0.002–0.003	500.00
A3	*P. varians*	II	−0.003, <0.001	13.99, 8.738–20.922	0.003, 0.003–0.003	333.33
A4	*P. varians*	II	−0.003, <0.001	16.53, 9.659–27.895	0.003, 0.003–0.003	333.33
A5	*P. varians*	II	−0.003, <0.001	15.80, 8.368–29.934	0.006, 0.006–0.006	166.67
A6	*P. varians*	II	−0.003, <0.001	19.31, 11.744–218.21	0.003, 0.003–0.004	333.33
A7	*P. varians*	II	−0.003, <0.001	11.68, 8.333–16.677	0.004, 0.004–0.005	250.00
A8	*P. varians*	II	−0.003, <0.001	8.58, 6.071–12.013	0.005, 0.005–0.006	200.00
A1	*A. salina*	II	−0.003, <0.001	16.87, 10.661–26.089	0.002, 0.002–0.003	500.00
A2	*A. salina*	II	−0.008, <0.001	11.06, 8.340–14.039	0.001, 0.001–0.001	1250.00
A3	*A. salina*	II	−0.010, <0.001	22.43, 11.876–30.684	0.001, 0.001–0.002	1000.00
A4	*A. salina*	II	−0.011, <0.001	30.51, 13.596–50.512	0.001, 0.001–0.002	1000.00
A5	*A. salina*	II	−0.003, <0.001	18.99, 9.833–195.687	0.003, 0.003–0.003	333.33
A6	*A. salina*	II	−0.006, <0.001	17.00, 11.752–37.074	0.001, 0.001–0.002	1000.00
A7	*A. salina*	II	−0.004, <0.001	13.14, 9.483–20.397	0.002, 0.002–0.002	500.00
A8	*A. salina*	II	−0.004, <0.001	12.25, 8.969–15.890	0.002, 0.002–0.002	500.00

**Figure 5.**
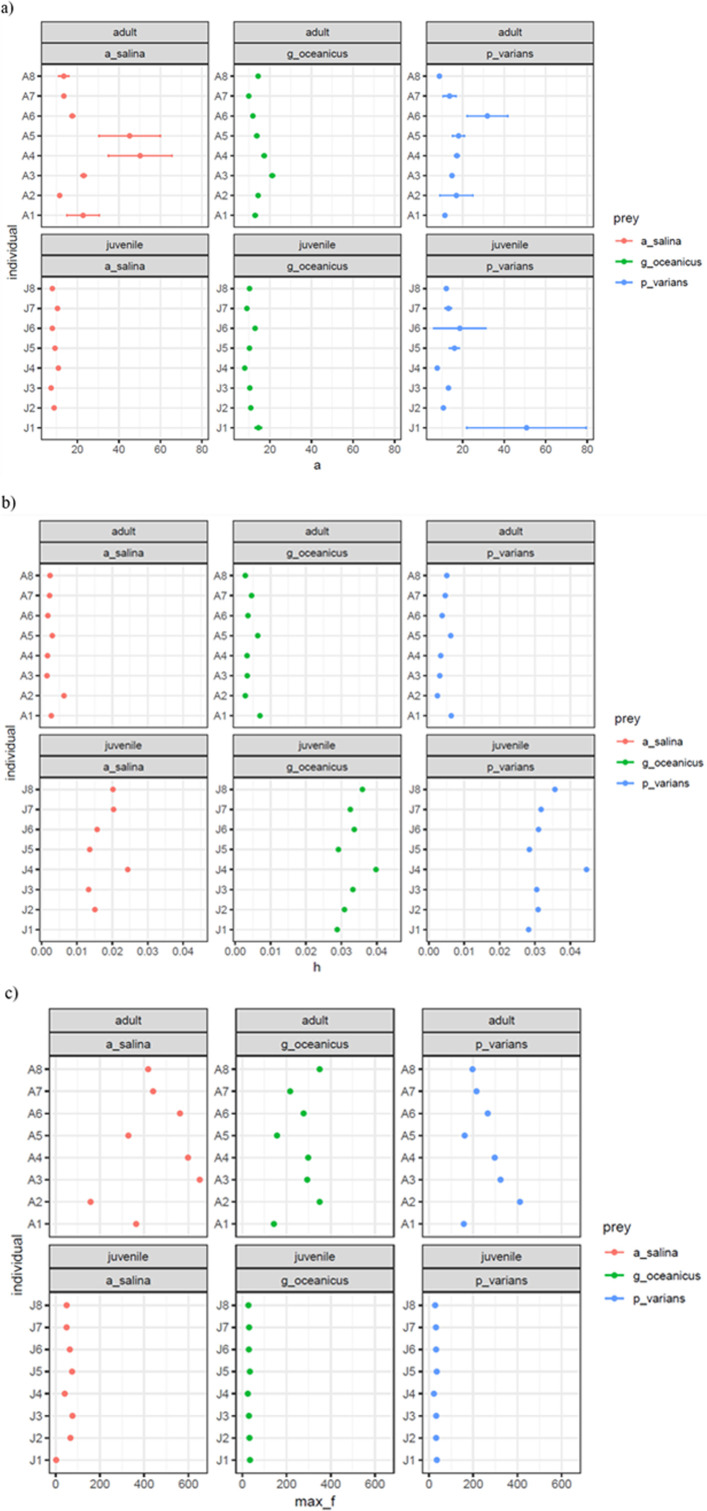
Mean (± SE) a) attack rate (a)*,* (b) handling time *h* and (c) maximum feeding rate 1 */h* derived from bootstrapping (*n* = 2000) of both juvenile and adult lionfish feeding towards all three prey types: *Artemia salina* (red), *Gammarus oceanicus* (green) and *Palaemonetes varians* (blue).

Feeding parameter estimates revealed distinct ontogenetic and prey-specific differences (table 6 and 7; figures 3 and 4). Adult lionfish exhibited consistently higher attack rates (*a*) than juveniles across all prey types, with the highest mean attack rate observed for *A. salina* (24.66 ± 1.51) and the lowest for *G. oceanicus* (14.38 ± 0.18). Juvenile lionfish had notably lower attack rates, particularly with *A. salina* (8.96 ± 0.08; see figure 5).

Handling time (*h*) was substantially shorter in adults than juveniles for all prey species. Adults had the shortest mean handling time when feeding on *A. salina* (0.00272 ± 0.00005), while juvenile lionfish took considerably longer, especially with *G. oceanicus* and *P. varians* (0.03301 ± 0.00013 s and 0.03261 ± 0.00018, respectively). As a result, maximum feeding rate (1 */h*) was markedly higher in adults. Adults reached the highest rate with *A. salina* (440.17 ± 5.37), while juveniles had the lowest with *G. oceanicus* (30.63 ± 0.11). Overall, both prey identity and life stage significantly influenced feeding efficiency, with *A. salina* producing the most favourable feeding parameters for both stages, though adults were markedly more efficient across all prey types.

At the individual level, FR magnitude varied most at higher prey densities. Among juveniles, individual J1 consistently showed the highest maximum feeding rates across all prey, while J4 had the lowest, indicating strong intra-stage variability. Similarly, adult A2 exhibited the highest maximum feeding rate across all prey types, but the lowest rates were prey-specific: A1 for *G. oceanicus* and *P. varians*, and A5 for *A. salina*. These results highlight the combined effects of ontogeny, prey identity and individual variability on lionfish feeding dynamics.

## 4. Discussion

Personalities play a crucial role in shaping an individual’s behaviour and responses to environmental challenges. In this study, the focus was on assessing personality differences between juvenile and adult lionfish to determine if these differences accounted for variations in feeding impacts. This study represents the first known investigation that examines the combination of FR and personality parameters in juvenile and adult lionfish, incorporating a novel object and shelter use. Our findings reveal the presence of personality within the context of lionfish behaviour, consistently observed across individuals. Surprisingly, there was no correlation between boldness and the maximum feeding rate, which was used as a proxy for impact. Although individuals displayed repeated bold behaviour, it did not necessarily translate to consuming the most food or having the highest impact. However, we did observe a connection between personality and reaction time in lionfish, which may be attributed to the vigilance levels exhibited by lionfish towards their prey. These findings suggest that lionfish personalities are not fixed, but are context-dependent, influenced by the social environment. Therefore, our initial hypothesis regarding the presence of a personality in lionfish was supported. However, there was no correlation found between boldness and predatory impact.

Heightened FR of invasive alien species are themselves a predictor of high ecological impact [25,54–56]. Based on observations from previous FR studies, the impact of lionfish is overwhelming [42,43,57–59]. While this study did not find a connection between personality and the impact measured through maximum feeding rates in FR it did produce FR values for each individual lionfish with varying results of impact. Context dependencies of behaviour can alter interspecific interaction strengths in a variety of settings. For example, noise pollution altered the behaviour of European minnows (*Phoxinus phoxinus*), leading to significant changes in FR and decreased attack rates [60].

The integration of FR analysis with investigations of personality is becoming increasingly common as ecologists seek to understand intraspecific variation in ecological niches [61–63]. More recently, McGlade et al. [64] successfully demonstrated the presence of behavioural syndromes (correlations of different behavioural traits) through novel object experiments involving rainbow and brown trout. Similarly, McGlade *et al*. [64] were unable to definitively predict impact based on personality types.

Since their invasion, lionfish have gained a reputation for their voracious appetite, but little is known about whether all lionfish feed in the same manner and to the same extent. There have been limited studies that incorporate assessments of feeding impact with behavioural analyses [61,65], and none have been conducted to date specifically on lionfish. Studies have shown that bold and risk-taking individuals are typically associated with exploratory behaviour, whereas shyer individuals tend to be more risk-averse and passive [15]. However, the interpretation of these traits can vary depending on environmental contexts, as individuals become familiar with foraging patches and adjust their behaviours, potentially leading to changes in social structures [66]. This highlights the importance of gaining further insights into the personalities of more elusive invasive alien species like lionfish. Interestingly in this study, when comparing the individual personality assessments with those conducted in group settings, the personality of the lionfish changed. In groups of eight individuals, bolder lionfish exhibited shyer personalities, while shyer individuals became bolder, displaying increased exploration of the novel object. Additionally, shyer fish spent less time in shelter when in groups compared to when they were isolated. These findings highlight the interaction between personality and social context, suggesting that group dynamics can significantly influence individual lionfish personality.

Although the influence of group dynamics on lionfish personality has not been extensively explored, the current study shows that both juvenile and adult lionfish exhibit consistent personalities when isolated, with their behaviour undergoing notable changes in group settings. In particular, shy fish became more explorative (bolder), while bolder fish exhibited reduced exploration. Similar shifts in personalities were observed in a study by Zhou *et al*. [67] on invasive mosquitofish (*Gambusia holbrooki*), where changes in environmental salinity altered group dynamics and led to shyer individuals becoming more prominent in social interactions. Previous studies have demonstrated populations with a wide range of personality traits are more prone to becoming invasive and rapidly spread [68,69]. In natural settings, differences in behaviour among individuals tend to be more consistent, but in laboratory conditions, these behaviours often disappear [70]. However, in the case of lionfish, their distinct personalities were evident when in the presence of other individuals. Although a change in personality was observed in group settings, the underlying mechanism for this phenomenon still requires a more comprehensive understanding. This study represents the first known instance of such behaviour in lionfish and could potentially be explained by the ‘audience effect’ [71]. The presence of an audience or group of individuals may lead to a change in behaviour dynamics, where individuals adjust their behaviour accordingly [72]. Highlighted in previous studies when caring for offspring [73], competing between rivals [74], competing for mates [75] and aggression in groups across taxa (crustaceans: [76]; fish: [77] and insects: [74]). The findings are broadly in line with studies like these where individuals whose behaviour was elevated in the presence of others may act to advertise or reinforce their own control within a group dynamic to deter potential conflicts [78]. DeRoy *et al*. [79] demonstrated consistent behaviours among individual lionfish—showing that they could be trained in food reward activities. In the current study, it is therefore possible that the lionfish developed learnt responses to the novel object due to the high replication of individuals in the experiment. Therefore, future research should include a greater number of individuals over a longer time period.

The combination of general inquisitiveness towards a novel object and a reduced time spent taking refuge in a shelter was found to be an important set of personalities in lionfish. It was observed that bolder lionfish, characterized by these traits, may be more likely to be harvested or culled [80]. This can have unintended and counterproductive consequences, as harvest-driven traits may lead to unexpected outcomes [81]. For instance, the removal of invasive pumpkinseed sunfish (*Lepomis gibbosus*) by anglers resulted in changes in population size and size at sexual maturity [82]. If lionfish were to reach sexual maturity and reproduce earlier than usual, it would have significant implications. This could lead to higher population growth rates [26] and further amplify their invasive impact on local ecosystems. The larger population size resulting from early sexual maturity has the potential to outcompete native species for vital resources like food and habitat [25]. This competition for resources can have negative ecological consequences, potentially leading to the decline of native species populations. Additionally, early sexual maturity would provide lionfish with more time and opportunities to expand their range, posing a greater threat to biodiversity and ecological balance.

Human-mediated removals have proven to be the most effective method for controlling lionfish densities [83], with spearfishing being a commonly employed technique [26,84–86]. Rapid implementation of removal efforts is crucial for their successful eradication [41,87,88]. Spearfishing activities have been observed to induce behavioural changes in lionfish populations, such as shifting their feeding patterns to dusk and dawn [35], with similar though less pronounced effects reported in the Mediterranean, where culling showed only limited influence on lionfish behaviour [3].

Bolder individuals in lionfish populations are more likely to be harvested or culled compared to their shyer and risk-averse counterparts, who tend to avoid divers [81,89]. The harvesting of lionfish can lead to changes in their personality, which in turn can impact population dynamics. Specific personalities are associated with prey consumption and survival, and the removal of individuals possessing these personalities can influence the growth rate, size structure and genetic diversity of the population [89]. While harvesting effectively reduces lionfish densities, the selective targeting of bolder individuals may cause the population to shift towards a higher proportion of shyer or more risk-averse individuals as a survival strategy. This behavioural adaptation can have consequences for interactions between invasive and native species, potentially leading to cascading effects on ecosystem dynamics [90]. The observed differences in personality between juvenile and adult lionfish populations in this study may reflect variations in foraging behaviours found in natural environments [91]. These differences in traits within lionfish populations, both juvenile and adult, likely contribute to their impacts within invaded ranges.

While this study provides initial evidence for the role of individual differences and personality in an invasive species, further research is necessary to determine if these traits are consistent in natural populations and their variations alongside native counterparts. It is important to note that the lionfish used in this study originated from wild-caught individuals from the western Atlantic invaded range that were subsequently bred in captivity by a private breeder. Species identity (*P. volitans* versus *Pterois miles*) could not be verified at the time of experimentation, although retrospective dissections and genetic testing confirmed all individuals as *P. volitans*. However, retrospective dissections and genetic confirmation conducted 11 months post-experiment confirmed that all individuals were *P. volitans*. This verification supports that our findings are representative of *P. volitans* specifically, and interpretations should therefore be considered within the context of its invaded Atlantic range. It can be hypothesized that the more exploratory and bold lionfish may be the ones driving the spread of invasions at the forefront. Assessing boldness within lionfish populations is particularly important for effective management. If bolder individuals are targeted and removed through culling methods, their impact is likely to be reduced. However, this leaves behind shyer lionfish individuals that are more challenging to capture, providing them with more time to reproduce and repopulate. Understanding the influence of changes in personality on invasive alien species is crucial for developing effective management strategies and predicting population responses. Incorporating knowledge of personality into invasive species management plans can enhance the sustainability and effectiveness of control or eradication efforts. Further investigations are needed to expand our understanding of how personality contribute to invasion success and to refine management approaches accordingly.

## Data Availability

The data is available from Dryad repository at [92]. Supplementary material is available online [93].
